# Functional evaluations comparing Billroth I with a large remnant stomach and Roux en Y with a small remnant stomach following laparoscopic distal gastrectomy for gastric cancer: An investigation including laparoscopic total gastrectomy

**DOI:** 10.1007/s00595-022-02557-w

**Published:** 2022-08-01

**Authors:** Eiji Nomura, Takatoshi Seki, Yamato Ninomiya, Hideki Izumi, Soichiro Yamamoto, Kazuhito Nabeshima, Kenji Nakamura, Masaya Mukai, Hiroyasu Makuuchi

**Affiliations:** 1 Department of Gastroenterological and General Surgery, Tokai University Hachioji Hospital, 1838 Ishikawa-machi, Hachioji, Tokyo 192-0032 Japan; 2 Department of Gastroenterological and General Surgery, Tokai University School of Medicine, 143 Shimokasuya, Isehara, Kanagawa 259-1193 Japan

**Keywords:** Gastric cancer, Laparoscopic distal gastrectomy, Billroth I reconstruction, Roux en Y reconstruction, Quality of life

## Abstract

**Purpose:**

This study compared the pros and cons of two post-distal gastrectomy (DG) reconstruction methods by comparing the patient quality of life and functional dynamics at one year postoperatively.

**Methods:**

We compared functional outcomes between Billroth I following laparoscopic 1/2 DG (L-B1; *n* = 27) and Roux en Y following laparoscopic 4/5 DG (L-RY; *n* = 24), including laparoscopic total gastrectomy (L-TG; *n* = 25), at one year postoperatively. Clinical investigations were performed in each patient, and functional evaluations by the acetaminophen (AAP) absorption test and plasma gastrointestinal hormone measurements were performed in consenting patients in each group (L-B1: *n* = 10, L-RY: *n* = 10, L-TG: *n* = 5).

**Results:**

Postoperative/preoperative body weight ratios were significantly higher in the L-B1 and L-RY groups, in descending order than the L-TG group, although the meal intake ratio was not significantly different between the L-B1 and L-RY groups. The incidence of remnant gastritis was significantly higher in the B1 than in the RY group. AAP levels, glucose and glucagon-like peptide 1 were significantly lower in the L-B1 than in the L-RY group. Active ghrelin levels (AGL) were similar between the L-B1 and L-RY groups.

**Conclusions:**

L-B1 maintains gradual intestinal absorption and physiological meal passage and prevents postoperative weight loss. L-RY results in maintenance of the postoperative meal intake via high AGL, equivalent to that in the L-B1 group.

## Introduction

Distal gastrectomy (DG) is the most commonly performed procedure for gastric cancer. Traditionally, the most common reconstruction methods that are performed after DG are the Billroth I (B1) and Billroth II (B2) procedures [1], although B2 tends to be avoided in Japan mainly due to the issue of subsequent bile reflux. Hence, the number of facilities that use the Roux en Y (RY) reconstruction method has been increasing [2]. For these reasons, many studies comparing the postoperative quality of life (QOL) between the B1 and RY methods have been reported recently, which have indicated a lack of a difference in the QOL between these methods [3–6].

However, since the B1 method has the benefit of involving only a single anastomosis, and the RY method is associated with reduced anastomotic tension although it causes meal stasis when the residual stomach is large, many facilities use the B1 method when the residual stomach is relatively large and RY reconstruction when the residual stomach is small [7]. In addition, it is difficult to definitively determine the superiority or inferiority of each reconstruction method. Previously, we compared the postoperative QOL one year after open DG in terms of the size of the remnant stomach and the reconstruction method and reported that the postoperative QOL was not related to the reconstruction method but rather was determined by the size of the remnant stomach [7, 8]. However, when considering the further long-term QOL, it is necessary to understand the changes in small intestinal absorption and hormone secretion kinetics caused by each meal intake as well as their combined effects on the long-term QOL [9].

Since operations for early gastric cancer have recently been performed laparoscopically, we evaluated changes in small intestinal absorption and hormone secretion dynamics with B1 and RY reconstruction following laparoscopic DG (LDG) one year after surgery in this study.

For early gastric cancer in the middle to lower a third of the stomach, when 1/2–2/3 of the stomach is resected with a safety margin of ≥ 2 cm, B1 reconstruction is typically performed, whereas for early gastric cancer in the upper to the middle third of the stomach in which 2/3–4/5 of the stomach is resected with a similar safety margin, RY reconstruction is performed.

For patients with severe esophageal hiatal hernia, we usually perform RY reconstruction with 2/3–4/5 DG to prevent reflux esophagitis, even in patients in whom it might otherwise have been possible to preserve a greater extent of the remnant stomach [10, 11]. We previously compared the QOL after open 2/3 DG with B1 versus that with RY and found that the QOL was almost the same between the two procedures [7, 8]. Therefore, in the present study, we excluded patients who underwent 2/3 DG and only included and investigated patients who underwent laparoscopic 1/2 DG with B1 (L-B1) and 4/5 DG with RY reconstruction (L-RY) as extreme cases.

The present study clarified and compared the pros and cons of L-B1 and L-RY by assessing the patients’ postoperative QOL and changes in absorptive and hormonal dynamics one year postoperatively.

## Patients and methods

This study included patients who underwent LDG and D1 + lymph node dissection from April 2015 to March 2020 at our hospital and were diagnosed preoperatively as cStage IA, IB and IIA (T3, N0), as such patients were not expected to receive anticancer drugs postoperatively, allowing us to largely eliminate their confounding negative effects on the QOL. Twenty-seven patients who underwent laparoscopic resection of the distal 1/2 of the stomach with B1 reconstruction using a delta-shaped anastomosis [12] (L-B1 group) and 24 patients who underwent laparoscopic distal 4/5 resection of the stomach with RY reconstruction [13] (L-RY group) were prospectively compared. In addition, 25 patients who underwent laparoscopic total gastrectomy with RY reconstruction (L-TG) during the same time period were used as the control group.

In our institute, each operator is expected to measure the ratio of the size of the resected stomach to the whole stomach and record it intraoperatively. In practice, they estimate the approximate size, classifying it as 1/2, 2/3, 4/5, or sometimes as “other” (providing a description in the designated space) [7]. Cases recorded as 2/3 or “other” were excluded from this study.

First, the postoperative digestive function, measured by the postoperative/preoperative body weight ratio (BWR) and postoperative/preoperative meal intake ratio (MIR), was determined in each patient. The MIR was estimated as the mean postoperative total daily meal intake compared to the preoperative intake. The data were acquired at a single time point (12 months postoperatively) using an in-house questionnaire that was mailed to the patients to avoid the potential influence of the researchers [7] (Table 1). The subjects completed the questionnaires and returned them to the researchers. Subsequently, endoscopic examinations and gastric emptying tests using acetaminophen (AAP) were performed.

**Table 1:** Questionnaire survey about postoperative body weight, meal intake and abdominal symptoms

1	Please state your present body weight ———kg
2	Please put a circle around the number below that fits your present postoperative total daily meal intake amount compared to your preoperative total meal intake
	20%
	40%
	60%
	80%
	100%
	Other ———%
3	Please circle the number below that best describes the present abdominal symptoms that you experience frequently, especially those occurring after meals
	Borborygmi
	Abdominal pain
	Diarrhea
	Nausea and/or vomiting
	Sensation of abdominal fullness
	Abdominal discomfort
	Heart burn or reflux
	No symptoms

Reprinted from our previous study by Nomura et al. [7]

Endoscopies performed from 6 to 12 months postoperatively at our outpatient clinic were analyzed to investigate the incidence of remnant gastritis and esophagitis. Endoscopic findings of the gastric remnant were evaluated according to the “residue, gastritis, bile” classification [14], and esophagitis was evaluated by the Los Angeles classification [15]. The presence of gastritis, residue grade ≥ 2, or esophagitis Grade ≥ A was considered a finding of clinical significance.

Finally, functional evaluations were performed for patients who underwent regular follow-up at our hospital and who agreed to participate in the study from July 2017 to March 2019. The course of gastric emptying was investigated using AAP ingestion in 10 patients in the L-B1 group, 10 patients in the L-RY group, and 5 patients in the L-TG group. AAP is absorbed not in the stomach but in the duodenum or jejunum, through which it enters the blood stream [16]. For the test, patients swallowed an alimentary liquid (200 mL of Ensure liquid mix^®^; Meiji, Tokyo, Japan) containing 1.5 g of AAP while in the sitting position as the physiological posture at the time of meal intake, and the concentration of AAP in blood was measured every 15 min for 60 min [7, 17]. At the same time, plasma insulin, glucose, gastrin, glucagon-like peptide 1 (GLP-1), active ghrelin and inactive ghrelin levels were also measured.

This study protocol was approved by the Human Ethics Review Committee of Tokai University School of Medicine (Institutional Review Board number 17R051). Written, informed consent was obtained from each enrolled patient before study entry in accordance with the Declaration of Helsinki.

Clinicopathological findings of the gastric resections were recorded according to the Japanese Classification of Gastric Carcinoma, 3rd English edition [18].

Statistical analyses were performed using Student’s *t* test and the *χ*^2^ test. Multiple comparisons for parametric data were calculated using the Bonferroni/Dunn method. A *P* value of less than 0.05 was considered significant.

## Results

### Patients’ clinical characteristics

All patients completed the questionnaires on digestive function. Patient demographics stratified according to the surgical procedure are presented in Table 2. No significant differences among the three experimental groups undergoing different procedures were observed for any of the items, except in terms of tumor location with each procedure. On further follow-up, there was no evidence of recurrence two years after surgery in any of the patients.

**Table 2:** Patient demographics stratified according to the surgical procedures

	L-B1 (*n* = 27)	L-RY (*n* = 24)	L-TG (*n* = 25)	*P* value
Sex (male:female)	19:8	13:11	17:8	0.436
Age (years)	64.9 ± 10.6	67.2 ± 9.0	71.4 ± 9.7	0.326
Preop. hiatal hernia (+ : −)	5:22	4:20	3:22	0.805
Preop. esophagitis (+ : −)	1:26	0:24	0:25	0.399
Tumor location^a^				
U	0	3	15	< 0.001
M	3	18	10	
L	24	3	0	
Stage (cases)				
IA	25	16	16	0.382
IB	1	5	4	
IIA	1	1	2	
IIB	0	1	2	
IIIA	0	1	1	

*L-B1* laparoscopic Billroth I reconstruction, *L-RY* laparoscopic Roux en Y reconstruction, *L-TG* laparoscopic total gastrectomy, *Preop* preoperative, *U* upper third of the stomach, *M* middle third of the stomach, *L* lower third of the stomach

^a^There were significant differences between the three groups in terms of tumor location

### Surgical parameters and postoperative results

Surgical parameters and postoperative results are listed in Table 3. The operative time was significantly shorter in the L-B1 group than in the L-RY and L-TG groups. This might have been mainly caused by differences in the number of anastomoses in each procedure. Furthermore, the postoperative hospital stay was significantly longer in the L-TG group than in the L-B1 group, probably due to the occurrence of postoperative complications for which the recovery time was long, such as pancreatic fistula.

**Table 3:** Surgical parameters and postoperative results

Op method Characteristics	L-BI	L-RY	L-TG	*P* value
Operative duration (min)	278.3 ± 50.4^a^	345.0 ± 49.0^a^	354.0 ± 48.3^a^	< 0.001
Blood loss (ml)	52.6 ± 104.8	80.9 ± 110.2	108.5 ± 147.2	0.262
Postoperative hospital stay (days)	10.8 ± 2.7^b^	12.7 ± 4.8	14.7 ± 7.1^b^	0.026
Postoperative complications (number)				
Anastomotic leakage	0	2	0	0.184
Pancreatic fistula	0	1	3	
Hemorrhage	0	0	1	
Anastomotic stenosis	1	0	1	

*Op* operative, *L-B1* laparoscopic Billroth I reconstruction, *L-RY* laparoscopic Roux en Y reconstruction, *L-TG* laparoscopic total gastrectomy

^a^There was a significant difference in operative duration between the L-B1 and L-RY/L-TG groups

^b^There was a significant difference in the duration of postoperative hospital stay between the L-B1 and L-TG groups

### Clinical outcomes at 12 months

**Fig. 1:**
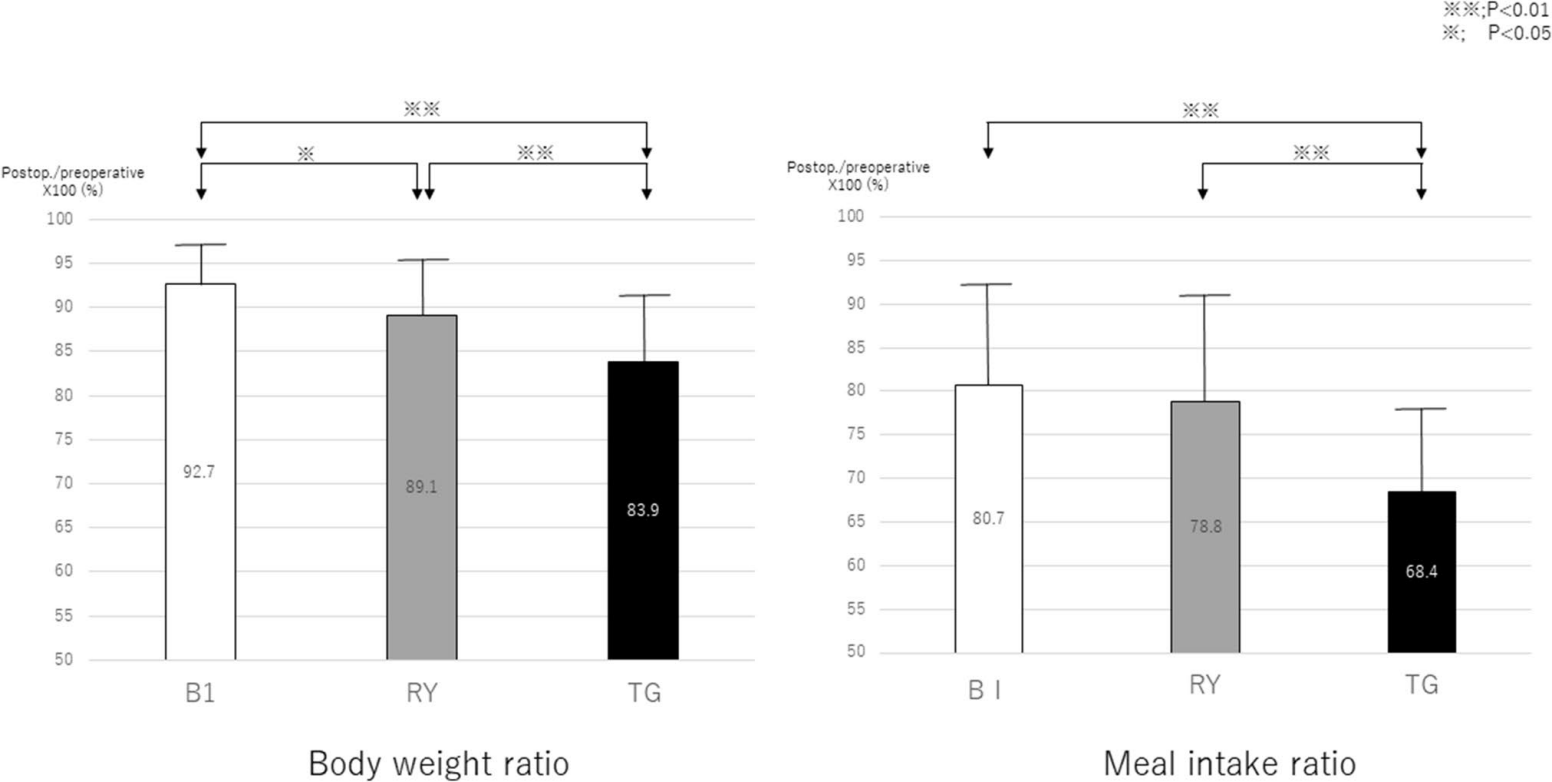
The postoperative/preoperative body weight ratio (BWR) and meal intake ratio (MIR) (%). The BWR was the highest in the L-B1 group, followed by the L-RY and then the L-TG group. The MIR was not significantly different between the L-B1 and L-RY groups

The BWR was the highest in the L-B1 group, followed by the L-RY group and then the L-TG group, with the differences between them being significant (Fig. 1). The MIR was significantly higher in the L-B1 and L-RY groups than in the L-TG group, but there was no significant difference between the L-B1 and L-RY groups.

A sensation of abdominal fullness was frequent in the L-B1 group (22.2%, 6/27), abdominal pain (25.0%, 6/24) was frequent in the L-RY group, and nausea (40.0%, 10/25) was frequent in the L-TG group, although there were no significant differences among the groups in terms of these complaints (Table 4). There were no cases of Petersen’s hernia or Roux stasis syndrome in the L-RY group.

**Table 4:** Abdominal symptoms and endoscopic findings

	L-B1	L-RY	L-TG	*P* value
Abdominal symptoms (number)				
Borborygmi	4	4	5	0.807
Abdominal pain	3	6	5	
Diarrhea	2	4	7	
Nausea/vomiting	4	3	10	
Abdominal fullness	6	4	3	
Abdominal discomfort	3	4	4	
Heart burn/Reflux	3	2	3	
No Symptoms	5	5	3	
Endoscopic finding [number %)]				
Reflux esophagitis	4 (14.8)	0 (0)	1 (4.0)	0.084
Remnant gastritis	8 (29.6)^a^	1 (4.2)^a^	–	0.017
Gastric residue	11 (40.7)^b^	0 (0)^b^		< 0.001

*L-B1* laparoscopic billroth I reconstruction, *L-RY* laparoscopic roux en Y reconstruction, *L-TG* laparoscopic total gastrectomy

^a^There was a significant difference in the incidence of remnant gastritis between the L-B1 and L-RY groups

^b^There was a significant difference in the incidence of residual food in the remnant stomach between the L-B1 and L-RY groups

### Endoscopic examination findings

The incidence of reflux esophagitis on an endoscopic examination was 14.8% (4/27) in the L-B1 group, 0% (0/24) in the L-RY group and 4.0% (1/25) in the L-TG group, although the differences among the groups were not significant. The incidence of remnant gastritis on an endoscopic examination was 29.6% (8/27) in the L-B1 group and 4.2% (1/24) in the L-RY group, indicating a significantly higher incidence in the L-B1 than in the L-RY group (*P* = 0.017). In particular, residual food in the remnant stomach (grade ≥ 1) was observed in 40.7% (11/27) of patients in only the L-B1 group and none of the patients in the L-RY group (*P* < 0.001) (Table 4).

### Functional outcomes at 12 months

**Fig. 2:**
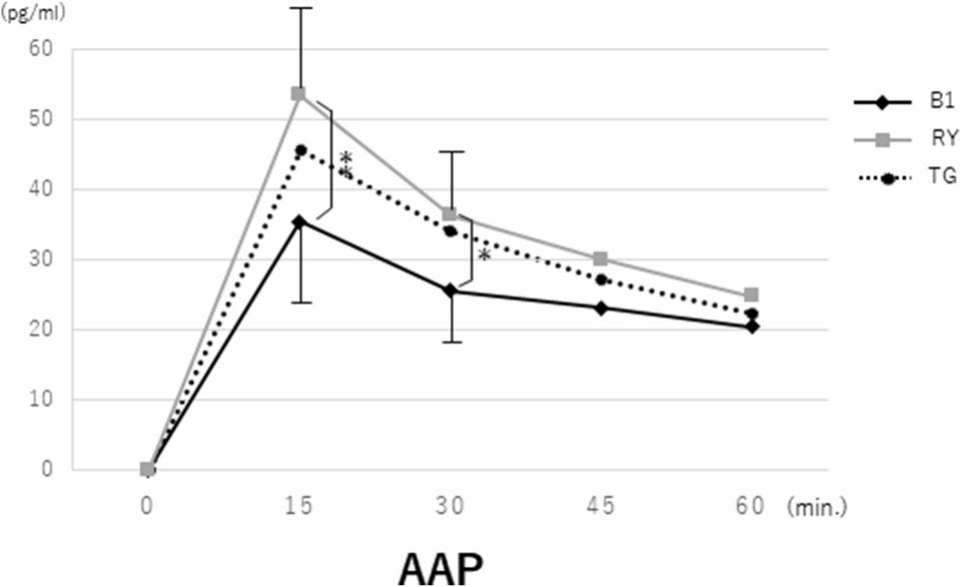
Changes in acetaminophen (AAP) concentrations in the sitting position. Plasma AAP concentrations in the L-RY group increased markedly 15 and 30 min after oral administration and were similar to those in the L-TG group, while the increase in the L-B1 group was gradual

The emptying test using AAP demonstrated that plasma AAP concentrations in the L-RY group increased markedly 15 and 30 min after oral administration and were close to but slightly higher than those in the L-TG group, with no statistical significance in the difference, whereas the increase in the L-B1 group was gradual; the differences between the L-B1 and L-RY groups were significant (Fig. 2). The time courses of glucose levels and GLP-1 levels in the three groups were similar to those of AAP levels, and significant differences between the L-B1 and L-RY groups were similarly observed at 15 and 30 min for GLP-1, although they were observed at later phases (30, 45 and 60 min) for glucose.

**Fig. 3:**
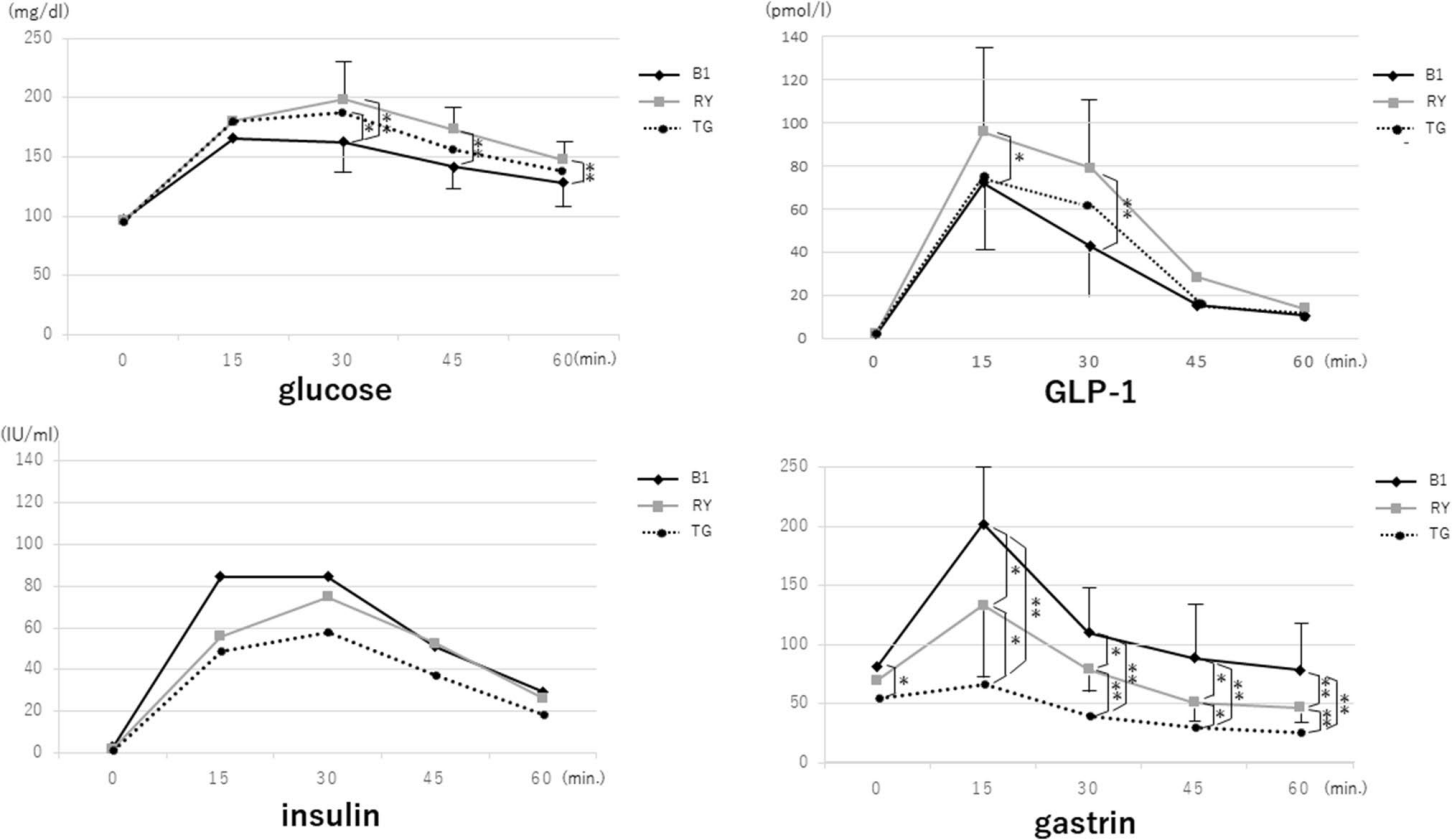
Changes in hormone levels in the sitting position. The patterns of glucose levels and GLP-1 levels in the three groups were similar to those of AAP levels, with significant differences being observed between the L-B1 and L-RY groups. Gastrin levels were higher in the L-B1 group than in the other groups

**Fig. 4:**
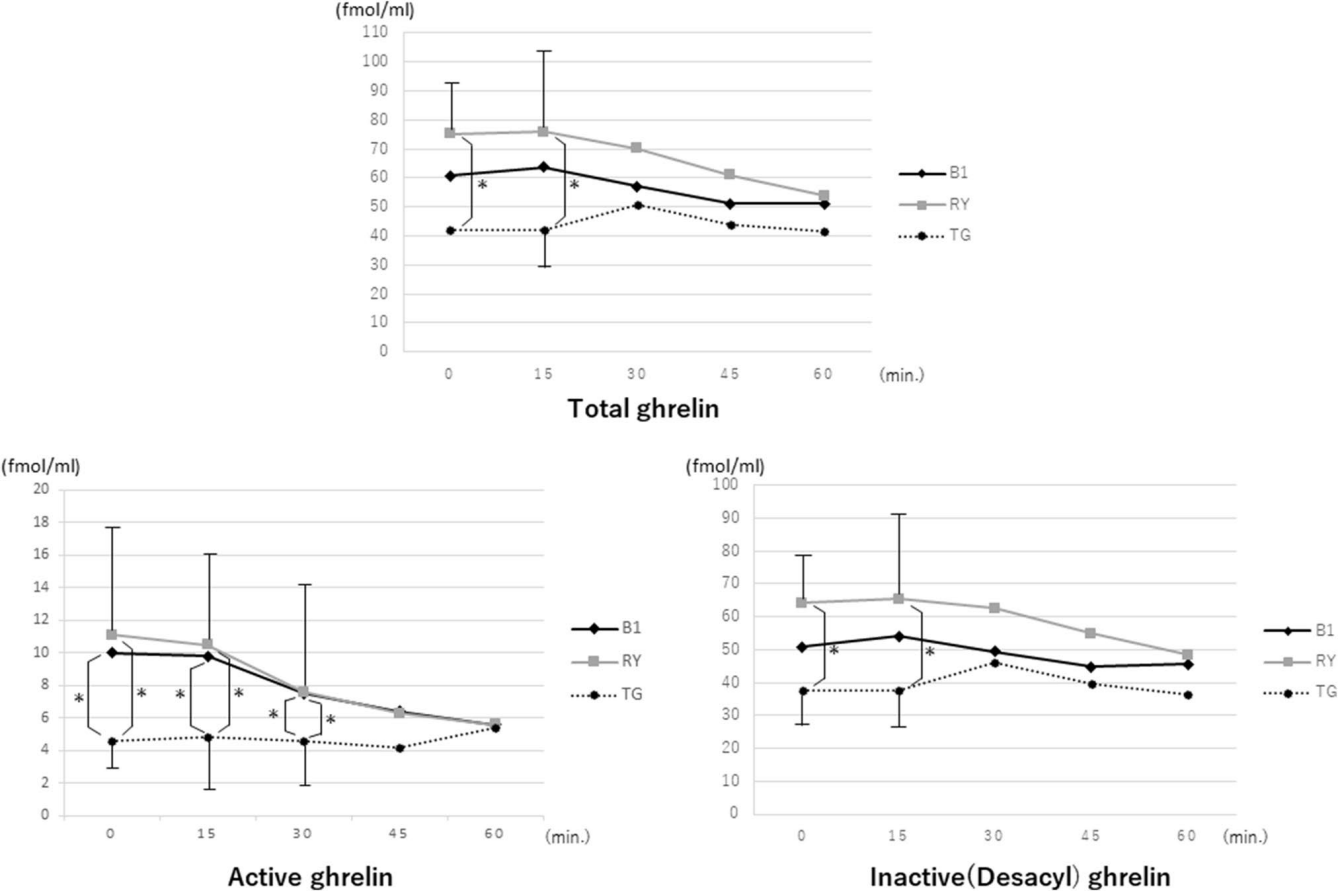
Changes in ghrelin levels in the sitting position. Since inactive ghrelin accounted for most of the measured ghrelin level, especially in the L-RY group, active ghrelin levels were almost the same between the L-B1 and L-RY groups

The levels of both gastrin and insulin secretion were higher in the L-B1 group than in the other groups. Furthermore, significant differences in gastrin levels among the three groups were observed at every measurement point, although there were no significant differences in insulin secretion among the three groups (Fig. 3). Total ghrelin levels in the L-RY group, however, were higher than those in the L-TG group. Since inactive ghrelin accounted for most of the measured ghrelin, especially in the L-RY group, active ghrelin levels were almost the same between the L-B1 and L-RY groups (Fig. 4).

## Discussion

Many reports have compared the patient QOL after RY versus B1 reconstruction following DG for gastric cancer, although detailed information on the size of the remnant stomach and minute reconstruction procedures have been ignored thus far. Although performing large-scale studies, including patients treated by various operations, is important for improving data reliability, such studies are insufficient for devising new reconstruction procedures that might improve the postoperative QOL.

The present study compared the postoperative QOL and gastrointestinal function between B1 and RY in terms of the size of the remnant stomach following L-DG with D1 + lymph node dissection at one year postoperatively. In this study, patients in whom the remnant stomach was half that of the preoperative size underwent reconstruction by B1, and cases with a remnant stomach 1/5 the size of the original stomach underwent reconstruction by the RY procedure. In addition, this study enrolled patients with early gastric cancer of Stage IA/IB/IIA (T3, N0) to exclude the influence of postoperative chemotherapy. Furthermore, since we felt that a comparison between only B1 and RY reconstruction might be insufficient, TG reconstruction, which usually results in the poorest QOL, was used as a control group [19, 20]. The study results showed that the BWR in the L-B1 group was significantly higher than in the L-RY and L-TG groups. Furthermore, the BWR in the L-RY group was significantly higher than that in the L-TG group. As we previously reported, the comparison of the BWR and MIR revealed better functional preservation among patients with a larger remnant stomach following open DG with D2 lymph node dissection, regardless of the type of reconstruction, than those with a smaller one [7].

In another study, we also examined whether or not the correlation between the BWR and MIR was a good indicator of the QOL and whether the absorptive kinetics of the small intestine could be expressed by the AAP concentration. That evaluation revealed a negative correlation between the AAP concentration at 15 min and BWR in all patients and a weak negative correlation between the AAP concentration at 15 min and MIR [17]. More specifically, there was some correlation between the slow intestinal absorption in the early postprandial phase and maintenance of the postoperative body weight and meal intake. However, since the AAP concentration was measured in the sitting position as the physiological posture at meal intake in this study, we did not observe any correlation between intestinal absorption and the postoperative body weight or meal intake. Meals are moved downwards mainly by peristaltic intestinal motion in the supine position and by both peristaltic and gravitational intestinal motion in the sitting position. As a result, the AAP curve is shifted upward from its location in the supine position to that in the sitting position. The upward shift of the AAP curve might have been caused by gravitational intestinal motion in the sitting position. Taken together, the present and previous findings show that a large remnant stomach leads to the maintenance of body weight postoperatively.

Furthermore, the evaluation of the dynamics of gastric hormone secretion indicated that the postprandial increase in blood sugar levels was suppressed in the L-B1 group compared to the L-RY group, as with the changes in AAP levels, showing that gastric emptying was gradual in the L-B1 group compared to the L-RY group in this study.

The maintenance of gastrin levels in the L-B1 group compared to the L-RY group indicated the extent to which the gastrin secretion area remained in the L-B1 group. The results of the evaluation of gastric hormonal levels suggest the need to retain as large a size of the remnant stomach as possible. However, our findings that insulin levels were higher in the L-B1 than the RY group, albeit not to a significant degree, and that GLP-1 levels in the L-RY group were significantly higher than those in the L-B1 group might reflect the effects of whether or not the meal passes through the duodenum. This is because blood sugar levels in the L-B1 group might be controlled by insulin that is stimulated by the glucose-dependent insulinotropic polypeptide (GIP) derived from K cells of the duodenum. Furthermore, blood sugar levels in the L-RY group might be mainly controlled by GLP-1. Chen et al. [21] noted an increased casual plasma GLP-1 level in patients who underwent RY reconstruction and a decreased ghrelin levels in patients who underwent B1 reconstruction after gastrectomy. Shoda et al. [22] state that their results support two hypotheses: the upper and lower intestinal hypotheses. The upper intestinal hypothesis posits that excluding the upper intestine may decrease the stimulation of K cells and ghrelin levels, thereby suppressing insulin counter-regulatory hormones and potentially leading to decreased glucose levels [23]. The lower intestinal hypothesis proposes that expedited nutrient delivery to the lower intestine enhances excessive secretion of GLP-1, a hormone that promotes insulin secretion [24], although insulin secretion in the RY group was not high in this study.

In addition, although total ghrelin levels were high in the RY group, inactive ghrelin accounted for most of it, and the levels of active ghrelin were the same in both groups. While there have been many reports about active ghrelin, details regarding the physiological actions of inactive ghrelin remain unknown [25, 26]. However, active ghrelin is well known as an acylated peptide that is produced predominantly in the upper part of the stomach and stimulates the appetite [27–29]. Our finding that the meal intake was significantly better maintained in the L-B1 and L-RY groups than in the L-TG group, albeit without significant differences observed between the L-B1 and L-RY groups, might be attributed to the active ghrelin levels rather than the action of inactive ghrelin. In other words, total ghrelin levels might have little effect on appetite following LDG, although a large meal intake might cause a slow increase in blood sugar levels owing to the reservoir function of the large remnant stomach.

Shinya et al. [30] reported that plasma ghrelin concentrations were lower and higher, respectively, in patients with simple obesity and anorexia nervosa. They also stated that ghrelin secretion is upregulated under conditions of negative energy balance and downregulated in the setting of positive energy balance, although only plasma des-acyl (inactive) ghrelin concentrations were measured in their study because of its stability. Thus, inactive ghrelin secretion in L-RY might be upregulated under conditions of more negative energy balance than that in L-B1 to maintain active ghrelin levels. A greater inactive ghrelin level might lead to a greater increase in active ghrelin levels due to the acylation of inactive ghrelin to active ghrelin. Furthermore, in L-TG cases, since it is difficult to upregulated ghrelin levels under conditions of negative energy balance for any reason [31], it is essential to preserve as much of the remnant stomach as possible.

A large remnant stomach and slow gastric emptying lead to the maintenance of the reservoir function. This reservoir function and emptying disorders are closely linked with each other. In the present study, meal residues were seen in 40.7%, and reflux esophagitis was recognized in 14.8% of subjects in the L-B1 group. Buhner et al. [32] reported that the remnant stomach does not usually contract after B1 gastrectomy in dogs and that duodenal contractile patterns influence gastric emptying. Mochiki et al. [33] reported on gastroduodenal motility after DG, stating that motor activity of the remnant stomach is inhibited and that interdigestive motor activity can be seen in the duodenum but not in the remnant stomach. Therefore, especially in patients with esophageal hiatal hernias, devices allowing maintenance of an appropriate size of the remnant stomach without stasis and reconstruction procedures, such as RY, are considered essential. Furthermore, the plasma AAP concentration in the TG group without a residual stomach was lower than that in the L-RY group, although the difference was not significant. As mentioned above, since AAP measurements in this study were performed in the sitting position, a small remnant stomach can be regarded as acting as a mere pipe through which fluid was likely to have been emptied promptly, unlike a solid meal. This small reservoir function in L-RY patients might make it possible for L-RY patients to eat and drink faster than L-TG patients.

Several limitations associated with the present study warrant mention, including the small sample size. The AAP method is primarily an indirect investigation of the gastric emptying function and uses only a liquid meal, even though patients usually consume solid meals. How solid meals are absorbed and metabolized remains unknown and the mechanisms need to be elucidated in a future study. Furthermore, although Takiguchi et al. [34] reported that the downregulation of plasma ghrelin by the intake of food is significantly greater in patients with vagus nerve preservation than in those with complete vagotomy, the effect of preservation of the vagus nerve was not assessed in this study. In addition, the role of inactive ghrelin still needs to be clarified. However, the assessment of AAP levels in the blood might enable the evaluation of the ease of intestinal absorption and status of hormone secretion following each operative method to a large extent. Further randomized clinical trials comparing L-B1 and L-RY, including those with the use of solid meals, will be needed to verify the various functions in detail. Furthermore, we should investigate the advantages of procedures that retain a larger remnant stomach, reconstruction methods that would allow physiological duodenal passage, devices that might prevent esophageal regurgitation, and the effects of preservation of the vagus nerve.

In conclusion, L-B1 maintains gradual intestinal absorption and physiological meal passage and prevents postoperative weight loss. L-RY results in the maintenance of the postoperative meal intake via high active ghrelin levels, equivalent to that in the L-B1 group, and has a low incidence of remnant gastritis. This may be the reason why L-RY is associated with a better QOL than L-TG, provided detailed dietary advice, such as the manner of meal intake, is followed. This study also reinforces the fact that as much of the remnant stomach as possible should be preserved to prevent the loss of energy homeostasis.

## Abbreviations

QOL: Quality of life
DG: Distal gastrectomy
TG: Total gastrectomy
LDG: Laparoscopic distal gastrectomy
B1: Billroth I reconstruction
B2: Billroth II reconstruction
RY: Roux en Y reconstruction
L-B1: Laparoscopic Billroth I reconstruction
L-RY: Laparoscopic Roux en Y reconstruction
L-TG: Laparoscopic total gastrectomy
BWR: Postoperative/preoperative body weight ratio
MIR: Postoperative/preoperative meal intake ratio
AAP: Acetaminophen
cStage: Clinical stage
GLP-1: Glucagon-like peptide 1
GIP: Glucose-dependent insulinotropic polypeptide
GHS-R: Growth-hormone secretagogue receptor
